# Supplementary material for: Hepatitis B virus seroepidemiology data for Africa: Modelling intervention strategies based on a systematic review and meta-analysis
Source: PLoS Med. 2020 Apr 21;17(4):e1003068. doi: 10.1371/journal.pmed.1003068 (PMC7173646; doi:10.1371/journal.pmed.1003068)
Supplement: S1 PRISMA Checklist — (PDF) [file pmed.1003068.s001.pdf]

PRISMA 2009 Checklist

Section/topic	#	Checklist item	Reported in section
**TITLE**			
Title	1	Identify the report as a systematic review, meta-analysis, or both.	Study title
**ABSTRACT**			
Structured summary	2	Provide a structured summary including, as applicable: background; objectives; data sources; study eligibility criteria, participants, and interventions; study appraisal and synthesis methods; results; limitations; conclusions and implications of key findings; systematic review registration number.	Study abstract
**INTRODUCTION**			
Rationale	3	Describe the rationale for the review in the context of what is already known.	Study Introduction paragraphs 4-5
Objectives	4	Provide an explicit statement of questions being addressed with reference to participants, interventions, comparisons, outcomes, and study design (PICOS).	Study Introduction paragraph 6
**METHODS**			
Protocol and registration	5	Indicate if a review protocol exists, if and where it can be accessed (e.g., Web address), and, if available, provide registration information including registration number.	Review protocol not published elsewhere
Eligibility criteria	6	Specify study characteristics (e.g., PICOS, length of follow-up) and report characteristics (e.g., years considered, language, publication status) used as criteria for eligibility, giving rationale.	Methods, 'HBV seroepidemiology for Africa' section
Information sources	7	Describe all information sources (e.g., databases with dates of coverage, contact with study authors to identify additional studies) in the search and date last searched.	Methods, 'HBV seroepidemiology for Africa' section
Search	8	Present full electronic search strategy for at least one database, including any limits used, such that it could be repeated.	Methods, 'HBV seroepidemiology for Africa' section
Study selection	9	State the process for selecting studies (i.e., screening, eligibility, included in systematic review, and, if applicable, included in the meta-analysis).	Methods, 'HBV seroepidemiology for Africa' section; Figure 1 (PRISMA flowchart)
Data collection process	10	Describe method of data extraction from reports (e.g., piloted forms, independently, in duplicate) and any processes for obtaining and confirming data from investigators.	Methods, 'HBV seroepidemiology for Africa' section
Data items	11	List and define all variables for which data were sought (e.g., PICOS, funding sources) and any assumptions and simplifications made.	Methods, 'HBV seroepidemiology for Africa' section
Risk of bias in individual studies	12	Describe methods used for assessing risk of bias of individual studies (including specification of whether this was done at the study or outcome level), and how this information is to be used in any data synthesis.	Methods, 'HBV seroepidemiology for Africa' section

Summary measures	13	State the principal summary measures (e.g., risk ratio, difference in means).	Methods, 'HBV seroepidemiology for Africa' section
Synthesis of results	14	Describe the methods of handling data and combining results of studies, if done, including measures of consistency (e.g., I^2) for each meta-analysis.	Methods, 'Statistical analysis of metadata'; 'Modelling the impact of adult vaccination vs. 'test and treat''

Section/topic	#	Checklist item	Reported on page #
Risk of bias across studies	15	Specify any assessment of risk of bias that may affect the cumulative evidence (e.g., publication bias, selective reporting within studies).	Methods, 'HBV seroepidemiology for Africa' section; Given the large number of studies involved in this review, this data was uploaded onto Figshare (open access) and cited in the text,https://figsharecom/s/4414fce1d474bc8a6198
Additional analyses	16	Describe methods of additional analyses (e.g., sensitivity or subgroup analyses, meta-regression), if done, indicating which were pre-specified.	Methods, 'Statistical analysis of metadata'; 'Modelling the impact of adult vaccination vs. 'test and treat''
**RESULTS**			
Study selection	17	Give numbers of studies screened, assessed for eligibility, and included in the review, with reasons for exclusions at each stage, ideally with a flow diagram.	Results, 'Studies identified through a systematic literature review'; Figure 1
Study characteristics	18	For each study, present characteristics for which data were extracted (e.g., study size, PICOS, follow-up period) and provide the citations.	Given the large number of studies involved in this review, this data was uploaded onto Figshare (open access) and cited in the text - https://figsharecom/s/4414fce1d474bc8a6198
Risk of bias within studies	19	Present data on risk of bias of each study and, if available, any outcome level assessment (see item 12).	Results, 'Quality appraisal of data identified through systematic literature review'; Figshare https://figsharecom/s/4414fce1d474bc8a6198
Results of individual studies	20	For all outcomes considered (benefits or harms), present, for each study: (a) simple summary data for each intervention group (b) effect estimates and confidence intervals, ideally with a forest plot.	(a) Summary data and quality assessments for each study on Figshare https://figsharecom/s/4414fce1d474bc8a6198 (b) Forest plots and CIs, Supplementary Figure 1
Synthesis of results	21	Present results of each meta-analysis done, including confidence intervals and measures of consistency.	Results, 'Prevalence of infection (HBsAg) is positively correlated with exposure (anti-HBc); Figure 3; Figure 5
Risk of bias across studies	22	Present results of any assessment of risk of bias across studies (see Item 15).	Results, 'Quality appraisal of data identified through systematic literature review'; Figshare https://figsharecom/s/4414fce1d474bc8a6198
Additional analysis	23	Give results of additional analyses, if done (e.g., sensitivity or subgroup analyses, meta-regression [see Item 16]).	Results, 'Prevalence of infection (HBsAg) is positively correlated with exposure (anti-HBc)';

			Figure 4
**DISCUSSION**			
Summary of evidence	24	Summarize the main findings including the strength of evidence for each main outcome; consider their relevance to key groups (e.g., healthcare providers, users, and policy makers).	Discussion, 'Insights into HBV epidemiology'; 'Insights into interventions'; ''Test and treat' in the context of HBV guidelines'; 'Implications for future investigation and practice changes'; Table 1
Limitations	25	Discuss limitations at study and outcome level (e.g., risk of bias), and at review-level (e.g., incomplete retrieval of identified research, reporting bias).	Discussion, 'Caveats and limitations'
Conclusions	26	Provide a general interpretation of the results in the context of other evidence, and implications for future research.	Discussion; All sections; Table 1
**FUNDING**			
Funding	27	Describe sources of funding for the systematic review and other support (e.g., supply of data); role of funders for the systematic review.	Footnote page, 'financial support'

From: Moher D, Liberati A, Tetzlaff J, Altman DG, The PRISMA Group (2009). Preferred Reporting Items for Systematic Reviews and Meta-Analyses: The PRISMA Statement. PLoS Med 6(7): e1000097. doi:10.1371/journal.pmed1000097

For more information, visit: **www.prisma-statement.org**.
