# Supplementary material for: Hepatitis B virus seroepidemiology data for Africa: Modelling intervention strategies based on a systematic review and meta-analysis
Source: PLoS Med. 2020 Apr 21;17(4):e1003068. doi: 10.1371/journal.pmed.1003068 (PMC7173646; doi:10.1371/journal.pmed.1003068)
Supplement: S1 Table — (PDF) [file pmed.1003068.s002.pdf]

S1 Table: Population data and HBV seroepidemiology for Uganda used to inform a model to determine impact of interventions. Further details of the model have been previously described [1].

Variable	Model Value (input)	Fitted by Model (output)	Literature Support	Reference	
HBV+ prevalence (HIV-)	10.30%	10.0% (95% CI 7.92-11.7)	10.30%	Bwogi et al., [2]	HBV prevalence
HBV+ prevalence (HIV+)	10.30%	10.0% (95% CI 7.92-11.7)	10.3%	**Presume same as for general population [2]. This assumption is previously reported [3] as well as being supported by current literature review.	
Anti-HBc prevalence (exposure seroprevalence)	42.0%	42.14% (95% CI 40.2-44%)	(Anti-HBc) – (HBsAg+ only) = 52.30% - 10.3%= 42%	Bwogi et al., [2]	
HIV prevalence in individuals >6 years of age in Uganda	Fixed at 6.5% (based on adult prevalence)	-----	6.5%	UNAIDS data for Uganda, [4]	
HIV prevalence in individuals age 1-6 years in Uganda	Fixed at 0.5%	-----	0.7%	Note no estimate in UNAIDS data. Conservative estimate used, based on Uganda AIDS indicator survey [5]	
HIV prevalence in individuals <1 years of age in Uganda	Fixed at 0.5%	-----	0.7%	Note no estimate in UNAIDS data. Conservative estimate used, based on Uganda AIDS indicator survey [5]	
Vertical transmission rate for HBV (HBeAg+)	free (uninformative prior)	80.4% (95% CI 71.9-88.4%)	70-80%	Gentile & Borgia [6]	HBV transmission
Vertical transmission rate for HBV (HBeAg-)	free (uninformative prior)	24.6% (95% CI 14.7-33.4%)	10-40%	Gentile & Borgia [6]	
HBeAg+ prevalence (within HBsAg+)	27%	26.9% (95% CI 24.8-29.0%)	27%	Matthews et al., [7]	
Clearance in individuals >6 years of age (or percent expected to have acute infection)	fixed at 95%	----	>95%	WHO HBV factsheet [8]	
Clearance in individuals <1 years of age (or percent expected to have acute infection)	Fixed at 15%	----	10-20%	WHO HBV factsheet, updated July 2016 [8]	
Clearance in individuals 1-6 years of age (or percent expected to have acute infection)	Fixed at 40%	----	30-50%	WHO HBV factsheet [8]	

Spontaneous clearance of chronic HBV	Free (uninformative prior)	0.31% (95% CI 0.37-0.71%)	0.73%; 1.15%; 2.26% annually	Ferreira et al., [9]; Chu et al., [10]; Lui et al., [11] (respectively)	
Duration of an acute infection	Fixed at 6 months	-----	6 months	Liang., [12]	
Rate of conversion from HBeAg+ to HBeAg-	Free (uninformative prior)	5.12% (95% CI 4.25-6.38%) across ages	0.8% a year for <5 yrs; 3% for 5-15 yrs; 8-15% for >15 yrs	Kao., [13]	
Life expectancy	Fixed at 63 years	-------	Males: 62.2yrs, Females: 64.2yrs	Uganda Bureau of Statistics, [14]	Demographics
Efficacy of vaccination against HBV infection if HIV- in ages <1, 1-6, >6 years	fixed at 95.2%, 89.2%, 79.6%	------	----	Estimated using this model and data from SA in previous study by McNaughton et al., [1]	Vaccine efficacy
Efficacy of vaccination against HBV infection if HIV+ in ages <1, 1-6, >6 years	fixed at 78.4%, 21.7%, 0.31%	-------	----	Estimated using this model and data from SA in previous study by McNaughton et al., [1]	

REFERENCES

1. McNaughton A, Lourenco J, Hattingh L, Adland E, Daniels S, van Zyl A, et al. HBV vaccination and PMTCT as elimination tools in the presence of HIV: insights from a clinical cohort and dynamic model. BMC Med. 2019;17:43. doi: 10.1186/s12916-019-1269-x.
2. Bwogi J, Braka F, Makumbi I, Mishra V, Bakamutumaho B, Nanyunja M, et al. Hepatitis B infection is highly endemic in Uganda: findings from a national serosurvey. Afr Health Sci. 2009;9(2):98-108. PubMed PMID: 19652743.
3. Matthews PC, Geretti AM, Goulder PJ, Klenerman P. Epidemiology and impact of HIV coinfection with hepatitis B and hepatitis C viruses in Sub-Saharan Africa. J Clin Virol. 2014;61(1):20-33. doi: 10.1016/j.jcv.2014.05.018. PubMed PMID: 24973812.
4. UNAIDS Estimates 2018. Country factsheets: Uganda. http://www.unaids.org/en/regionscountries/countries/uganda.
5. Uganda AIDS Indicator Survey (AIS); Ministry of Health, Kampala, Uganda. http://health.go.ug/docs/UAIS_2011_REPORT.pdf: 2011.
6. Gentile I, Borgia G. Vertical transmission of hepatitis B virus: challenges and solutions. Int J Womens Health. 2014;6:605-11. doi: 10.2147/IJWH.S51138. PubMed PMID: 24966696; PubMed Central PMCID: PMCPMC4062549.
7. Matthews PC, Beloukas A, Malik A, Carlson JM, Jooste P, Ogwu A, et al. Prevalence and Characteristics of Hepatitis B Virus (HBV) Coinfection among HIV-Positive Women in South Africa and Botswana. PLoS One. 2015;10(7):e0134037. doi: 10.1371/journal.pone.0134037. PubMed PMID: 26218239; PubMed Central PMCID: PMC4517770.
8. WHO. Hepatitis B Fact Sheet 2017; http://www.who.int/mediacentre/factsheets/fs204/en/.
9. Ferreira SC, Chacha SG, Souza FF, Teixeira AC, Santana RC, Villanova MG, et al. Factors associated with spontaneous HBsAg clearance in chronic hepatitis B patients followed at a university hospital. Ann Hepatol. 2014;13(6):762-70. PubMed PMID: 25332262.
10. Chu CM, Liaw YF. HBsAg seroclearance in asymptomatic carriers of high endemic areas: appreciably high rates during a long-term follow-up. Hepatology. 2007;45(5):1187-92. doi: 10.1002/hep.21612. PubMed PMID: 17465003.
11. Liu J, Yang HI, Lee MH, Lu SN, Jen CL, Wang LY, et al. Incidence and determinants of spontaneous hepatitis B surface antigen seroclearance: a community-based follow-up study. Gastroenterology. 2010;139(2):474-82. doi: 10.1053/j.gastro.2010.04.048. PubMed PMID: 20434450.
12. Liang TJ. Hepatitis B: the virus and disease. Hepatology. 2009;49(5 Suppl):S13-21. doi: 10.1002/hep.22881. PubMed PMID: 19399811; PubMed Central PMCID: PMCPMC2809016.
13. Kao JH. Role of viral factors in the natural course and therapy of chronic hepatitis B. Hepatol Int. 2007;1(4):415-30. doi: 10.1007/s12072-007-9033-2. PubMed PMID: 19669337; PubMed Central PMCID: PMCPMC2717552.
14. Uganda Bureau of Statistics. Statistical Report. https://ubos.org/wp-content/uploads/publications/03_20182017_Statistical_Abstract.pdf: 2017.
