# Supplementary material for: Hepatitis B virus seroepidemiology data for Africa: Modelling intervention strategies based on a systematic review and meta-analysis
Source: PLoS Med. 2020 Apr 21;17(4):e1003068. doi: 10.1371/journal.pmed.1003068 (PMC7173646; doi:10.1371/journal.pmed.1003068)
Supplement: S2 Table — These studies (n = 13) were each reported in a single publication but contribute ≥2 data points to our overall analysis. Differences in the cohorts are highlighted in city/location, cohort characteristics, and cohort size. Studies listed in alphabetical order by location. (PDF) [file pmed.1003068.s003.pdf]

S2 Table: Details of studies from Africa reporting HBV prevalence (prev.) data from ≥2 cohorts. These studies (n=13) were each reported in a single publication but contribute ≥2 data points to our overall analysis. Differences in the cohorts are highlighted in city/location, cohort characteristics and cohort size. Studies listed in alphabetical order by location.

Country	First author and reference	Citation	PMID or DOI	City / location	Cohort Characteristics	Size of cohort (n=)	Anti-HBc prev.	Anti-HBc (& HBsAg negative) prev.	HBsAg prev.	Estimated susceptible population (100% - anti-Hbc prev.)	Reason for multiple cohorts
**Burkina Faso**	Collenberg [1]	J Med Virol. 2006 May;78(5):683-92.	DOI: 10.1002/jmv.20593	Ouagadougou (urban)	Blood donors, antenatal	238	76.40%	59.10%	17.30%	23.60%	Urban (Ouagadougou) vs Rural (Nouna)
**Burkina Faso**				Nouna (rural)	Blood donors, antenatal	289	69.60%	55.30%	14.30%	30.40%	
**Ethiopia**	Shiferaw [2]	BMC Res Notes. 2011 Nov 3;4:479.	DOI: 10.1186/1756-0500-4-479	Addis Ababa	Medical waste handlers	126	47.60%	41.30%	6.30%	52.40%	Different healthcare workers
**Ethiopia**				Addis Ababa	Non medical waste handlers	126	31.70%	30.90%	0.80%	68.30%	
**Gambia**	Peto [3]	BMC Infect Dis. 2014 Jan 7;14:7	PMID: 24397793	Multiple sites	Vaccinated young adults	278	27.40%	26.60%	0.80%	72.60%	Vaccination status
**Gambia**				Multiple sites	Unvaccinated young adults	475	56.00%	43.60%	12.40%	44.00%	
**Mauritania**	Mansour [4]	Journal of Medical Virology 84:1186–1198 (2012)	DOI: 10.1002/jmv.23336	Nouakchott	Antenatal	1020	66.30%	55.60%	10.70%	33.70%	Different patient groups
**Mauritania**				Nouakchott	Patients	946	76.50%	58.20%	18.30%	23.50%	

**Nigeria**	Belo [5]	East Afr Med J. 2000 May;77(5):283-5.	PMID: 12858922	Lagos	Healthcare workers (surgeons)	167	61.70%	36.00%	25.70%	38.30%	Different healthcare workers
**Nigeria**				Lagos	Admin staff (hospital)	193	53.40%	38.40%	15.00%	46.60%	
**Nigeria**	Onyekwere [6]	Niger Postgrad Med J. 2002 Sep;9(3):129-33.	PMID: 12501266	Lagos	Diabetics	100	45.00%	25.00%	20.00%	55.00%	Different patient groups
**Nigeria**				Lagos	Outpatients	80	57.00%	39.50%	17.50%	43.00%	
**Reunion**	Michault [7]	Bull Soc Pathol Exot. 2000 Feb;93(1):34-40.	PMID: 10774493	Reunion	Antenatal	1455	6.35%	5.72%	0.63%	93.65%	Different populations
**Reunion**				Reunion	Prison	100	10.00%	8.00%	2.00%	90.00%	
**South Africa**	Mayaphi [8]	S Afr Med J. 2012 Feb 23;102(3 Pt 1):157-62.	PMID: 22380911	Tshwane District Hospital, Pretoria	HIV negative	200	3.50%	1.50%	2.00%	96.50%	HIV status
**South Africa**				Tshwane District Hospital, Pretoria	HIV positive	200	16.00%	9.50%	6.50%	84.00%	
**South Africa**	Mphahlele [9]	J Clin Virol. 2006 Jan;35(1):14-20.	DOI: 10.1016/j.jcv.2005.04.003	Medunsa Campus; near Pretoria	HIV positive	167	85.00%	49.80%	35.20%	15.00%	HIV status
**South Africa**				Medunsa Campus; near Pretoria	HIV negative	128	82.00%	65.80%	16.20%	18.00%	

**Togo**	Dorkenoo [10]	Med Sante Trop. 2014 Jul-Sep;24(3):266-70	DOI: 10.1684/mst.2014.0341	Lome	Vaccinated healthcare workers	100	68.00%	32.00%	36.00%	32.00%	Vaccination status
**Togo**				Lome	Unvaccinated students	50	88.00%	32.00%	56.00%	12.00%	
**Uganda**	Nakwagala [11]	East Afr Med J. 2002 Feb;79(2):68-72.	PMID: 12380879	Mulago	HIV positive	129	65.10%	47.30%	17.80%	34.90%	HIV status
**Uganda**				Mulago	HIV negative	129	41.90%	28.80%	13.10%	58.10%	
**Uganda**	Price [12]	AIDS. 2017, March 21,Price et al, epub	DOI: 10.1097/QAD.0000000000001454	Kampala and Entebbe	HIV positive	2317	52.00%	46.00%	6.00%	48.00%	Different countries
**Zimbabwe**				Harare	HIV positive	999	56.30%	39.60%	16.70%	43.70%	
**Benin**	Bronowicki et al. [13]	J Hepatol. 2008 Apr;48(4):532-9.	DOI: 10.1016/j.jhep.2007.11.017	Cotonou	Hospital trial, healthy volunteers	305	75.4%	67.55	7.9%	24.6%	Different countries
**Togo**				Lome	Hospital trial, healthy volunteers	150	83.3%	60.7%	22.7%	16.7%	

REFERENCES

1. Collenberg E, Ouedraogo T, Ganame J, Fickenscher H, Kynast-Wolf G, Becher H, et al. Seroprevalence of six different viruses among pregnant women and blood donors in rural and urban Burkina Faso: A comparative analysis. J Med Virol. 2006;78(5):683-92. PubMed PMID: 16555290.
2. Shiferaw Y, Abebe T, Mihret A. Hepatitis B virus infection among medical aste handlers in Addis Ababa, Ethiopia. BMC research notes. 2011;4:479. doi: 10.1186/1756-0500-4-479. PubMed PMID: 22051187; PubMed Central PMCID: PMCPMC3234303.
3. Peto TJ, Mendy ME, Lowe Y, Webb EL, Whittle HC, Hall AJ. Efficacy and effectiveness of infant vaccination against chronic hepatitis B in the Gambia Hepatitis Intervention Study (1986-90) and in the nationwide immunisation program. BMC Infect Dis. 2014;14:7. doi: 10.1186/1471-2334-14-7. PubMed PMID: 24397793; PubMed Central PMCID: PMCPMC3898092.
4. Mansour W, Malick FZ, Sidiya A, Ishagh E, Chekaraou MA, Veillon P, et al. Prevalence, risk factors, and molecular epidemiology of hepatitis B and hepatitis delta virus in pregnant women and in patients in Mauritania. J Med Virol. 2012;84(8):1186-98. doi: 10.1002/jmv.23336. PubMed PMID: 22711346.
5. Belo AC. Prevalence of hepatitis B virus markers in surgeons in Lagos, Nigeria. East Afr Med J. 2000;77(5):283-5. PubMed PMID: 12858922.
6. Onyekwere CA, Anomneze EE, Wali SS. Prevalence of serological markers of chronic hepatitis B virus infection in diabetics in the Lagos University Teaching Hospital, Lagos. The Nigerian postgraduate medical journal. 2002;9(3):129-33. PubMed PMID: 12501266.
7. Michault A, Faulques B, Sevadjan B, Troalen D, Marais A, Barau G. [Prevalence of hepatitis A, B, C virus markers in Reunion (south hospital and Saint Pierre prison)]. Bull Soc Pathol Exot. 2000;93(1):34-40. PubMed PMID: 10774493.
8. Mayaphi SH, Roussow TM, Masemola DP, Olorunju SA, Mphahlele MJ, Martin DJ. HBV/HIV co-infection: the dynamics of HBV in South African patients with AIDS. South African medical journal = Suid-Afrikaanse tydskrif vir geneeskunde. 2012;102(3 Pt 1):157-62. Epub 2012/03/03. PubMed PMID: 22380911.
9. Mphahlele MJ, Lukhwareni A, Burnett RJ, Moropeng LM, Ngobeni JM. High risk of occult hepatitis B virus infection in HIV-positive patients from South Africa. J Clin Virol. 2006;35(1):14-20. Epub 2005/05/27. doi: 10.1016/j.jcv.2005.04.003. PubMed PMID: 15916918.
10. Dorkenoo AM, Kolou M, Sawadogo H, Feteke L, Agbenu E, Issa SA, et al. [Hepatitis B virus serologic status among hospital health care staff in Lome]. Med Sante Trop. 2014;24(3):266-70. doi: 10.1684/mst.2014.0341. PubMed PMID: 24922616.
11. Nakwagala FN, Kagimu MM. Hepatitis B virus and hiv infections among patients in Mulago hospital. East Afr Med J. 2002;79(2):68-72. PubMed PMID: 12380879.
12. Price H, Dunn D, Zachary T, Vudriko T, Chirara M, Kityo C, et al. Hepatitis B serological markers and plasma DNA concentrations. AIDS. 2017;31(8):1109-17. doi: 10.1097/QAD.0000000000001454. PubMed PMID: 28328795; PubMed Central PMCID: PMCPMC5414544.
13. Bronowicki JP, Abdelmouttaleb I, Peyrin-Biroulet L, Venard V, Khiri H, Chabi N, et al. Methylenetetrahydrofolate reductase 677 T allele protects against persistent HBV infection in West Africa. J Hepatol. 2008;48(4):532-9. doi: 10.1016/j.jhep.2007.11.017. PubMed PMID: 18222012.
