# Supplementary material for: Hepatitis B virus seroepidemiology data for Africa: Modelling intervention strategies based on a systematic review and meta-analysis
Source: PLoS Med. 2020 Apr 21;17(4):e1003068. doi: 10.1371/journal.pmed.1003068 (PMC7173646; doi:10.1371/journal.pmed.1003068)
Supplement: S3 Table — WLR was performed using cohort size as weight. Predicted HBsAg prevalence by WLR for Northern, Eastern, Southern, Western, and Central Africa at anti-HBc prevalences ranging from 5% to 95%, increasing in increments of 5%. Values are plotted in S3 Fig. WLR, weighted linear regression. (PDF) [file pmed.1003068.s004.pdf]

S3 Table: Predicted HBsAg prevalence for Northern, Eastern, Southern, Western and Central Africa, based on a given anti-HBc prevalence.

Weighted linear regression (WLR) was performed using cohort size as weight. Predicted HBsAg prevalence by WLR for Northern, Eastern, Southern, Western and Central Africa at anti-HBc prevalences ranging from 5-95%, increasing in increments of 5%. Values are plotted in S3 Fig.

	**Predicted HBsAg prevalence (%) by African region**				
**Anti-Hbc Prevalence (%)**	**West**	**East**	**South**	**Central**	**North**
**5**	5.87	0	0	9.65	0.89
**10**	6.43	0.56	1.3	9.79	1.62
**15**	6.98	1.53	2.72	9.94	2.36
**20**	7.54	2.51	4.14	10.09	3.09
**25**	8.09	3.48	5.56	10.24	3.83
**30**	8.64	4.46	6.99	10.39	4.56
**35**	9.2	5.43	8.41	10.53	5.29
**40**	9.75	6.41	9.83	10.68	6.03
**45**	10.3	7.38	11.25	10.83	6.76
**50**	10.86	8.36	12.67	10.98	7.5
**55**	11.41	9.33	14.1	11.13	8.23
**60**	11.97	10.31	15.52	11.27	8.97
**65**	12.52	11.28	16.94	11.42	9.7
**70**	13.07	12.26	18.36	11.57	10.44
**75**	13.63	13.23	19.78	11.72	11.17
**80**	14.18	14.21	21.21	11.86	11.9
**85**	14.74	15.18	22.63	12.01	12.64
**90**	15.29	16.16	24.05	12.16	13.37
**95**	15.84	17.13	25.47	12.31	14.11
