# Supplementary material for: Hepatitis B virus seroepidemiology data for Africa: Modelling intervention strategies based on a systematic review and meta-analysis
Source: PLoS Med. 2020 Apr 21;17(4):e1003068. doi: 10.1371/journal.pmed.1003068 (PMC7173646; doi:10.1371/journal.pmed.1003068)
Supplement: S4 Table — (PDF) [file pmed.1003068.s005.pdf]

S4 Table: Results of a systematic literature review to identify evidence or recommendations for use of catch up HBV vaccination in adolescents and adults in Africa.

Manuscript title	First author & citation	Summary comments
Hepatitis B virus infections in apparently healthy urban Nigerians: data from pre-vaccination tests.	Adoga [1]	Reports prevalence of HBV infection and concludes that 'The Nigerian government hepatitis B vaccination programme, which hitherto is limited to the National Childhood Immunisation Programme, should include the adult population'.
Decreasing the hepatitis B burden in Tunisia need more attention to adults for vaccination.	Alavian [2]	This is a letter in response to Chaouch et al [3]. The authors suggest that infection is occuring in later childhood/adolescence, and advocate catch-up/adult vaccination.
Evidence for a change in the epidemiology of hepatitis B virus infection after nearly two decades of universal hepatitis B vaccination in South Africa.	Amponsah-Dacosta [4]	Reports wane of vaccine immunity, and the difference between HIV+ and HIV- groups. The authors raise the question 'whether the time has come to consider a pre-adolescence vaccine booster dose policy'.
[Study of factors influencing hepatitis B immunization coverage in 1 to 8-years-old children in the Ouidah health district in Benin in 2007].	Bossali [5]	Study of factors influencing HBV immunization coverage in children, mothers and healthcare workers. Finding that immunization coverage decreased with age, the authors advocate 'catch-up [vaccination] sessions....in high prevalence areas' (NB. authors do not define catch up in older children vs adolescents or adults).
Investigating hepatitis B immunity in patients presenting to a paediatric haematology and oncology unit in South Africa.	Buchner [6]	Investigation of seroepidemiology in children in a high risk group. 40% had no immunity to HBV despite presumed vaccination. Suggests 'consider booster vaccination to the population as a whole.'
An update after 16 years of hepatitis B vaccination in South Africa.	Burnett [7]	Concludes on need for infant vaccine coverage, introduction of birth dose vaccine, switch to hexavalent vaccine, and consider vaccination for 12 year olds (if not vaccinated as infants).
Hepatitis B infection is highly endemic in Uganda: findings from a national serosurvey.	Bwogi [8]	HBsAg prevalence 10%. Risk factors: poor, un-educated, uncircumcised, ethnic group, HIV+. Conclude: 'The hepatitis B infant immunization programme should be sustained and catch-up vaccination considered for older children'.
Impact and long-term protection of hepatitis B vaccination: 17 years after universal hepatitis B vaccination in Tunisia.	Chaouch [3]	HBV seromarkers were checked in students. Raises the question of whether boosters in adolescence should be implemented, but doesn't currently suggest either way whether this should be undertaken.
Molecular epidemiology of human liver cancer: insights into etiology, pathogenesis and prevention from The Gambia, West Africa.	Kirk [9]	Review article of HCC causes, molecular associations in the Gambia. Addresses the question of booster doses, though without reaching a firm conclusion.

Observational study of vaccine efficacy 24 years after the start of hepatitis B vaccination in two Gambian villages: no need for a booster dose.	Mendy [10]	Cross sectional serological surve to determine vaccine efficacy. Efficacy against infection was 85%. Study looks at evidence for booster dose vaccine and says not helpful.
Long-term protection against HBV chronic carriage of Gambian adolescents vaccinated in infancy and immune response in HBV booster trial in adolescence.	van der Sande [11]	Cross-sectional study in the Gambia. Vaccine efficacy 15 years after vaccination was 67% against infection (anti-HBc positivity), and 97% against active infection (HBsAg carriage). For boosted participants anti-HBs responses were 38 IU/l prior to vaccination, 524 IU/l two weeks after boosting, and 101 IU/l after 1 year.
Observational study of vaccine efficacy 14 years after trial of hepatitis B vaccination in Gambian children.	Whittle [12]	Cross-sectional serological study. Vaccine-mediated antibody concentration dipped in late teen years associated with breakthrough infections. Refers to natural boosting as a result of sexual exposure in adolesence rather than booster vaccination.
A systematic review of hepatitis B screening economic evaluations in low- and middle-income countries.	Wright [13]	Meta-analysis of 9 studies looking at screening effectiveness in low-middle income countries. Concludes that screening with catch up vaccination for yound adults was benecifial.

REFERENCES

1. Adoga MP, Gyar SD, Pechulano S, Bashayi OD, Emiasegen SE, Zungwe T, et al. Hepatitis B virus infections in apparently healthy urban Nigerians: data from pre-vaccination tests. Journal of infection in developing countries. 2010;4(6):397-400. PubMed PMID: 20601793.
2. Alavian SM. Decreasing the hepatitis B burden in Tunisia need more attention to adults for vaccination. Epidemiology and infection. 2017;145(7):1512. doi: 10.1017/S0950268817000061. PubMed PMID: 28202090.
3. Chaouch H, Hachfi W, Fodha I, Kallala O, Saadi S, Bousaadia A, et al. Impact and long-term protection of hepatitis B vaccination: 17 years after universal hepatitis B vaccination in Tunisia. Epidemiology and infection. 2016:1-11. doi: 10.1017/S0950268816001849. PubMed PMID: 27535719.
4. Amponsah-Dacosta E, Lebelo RL, Rakgole JN, Burnett RJ, Selabe SG, Mphahlele MJ. Evidence for a change in the epidemiology of hepatitis B virus infection after nearly two decades of universal hepatitis B vaccination in South Africa. J Med Virol. 2014;86(6):918-24. doi: 10.1002/jmv.23910. PubMed PMID: 24615635.
5. Bossali F, Paraiso M, Bokossa A, Fourn L. [Study of factors influencing hepatitis B immunization coverage in 1 to 8-years-old children in the Ouidah health district in Benin in 2007]. Med Trop (Mars). 2010;70(2):149-54. PubMed PMID: 20486350.
6. Buchner A, Omar FE, Vermeulen J, Reynders DT. Investigating hepatitis B immunity in patients presenting to a paediatric haematology and oncology unit in South Africa. South African medical journal = Suid-Afrikaanse tydskrif vir geneeskunde. 2014;104(9):628-31. doi: 10.7196/samj.7952. PubMed PMID: 25212405.

7. Burnett RJ, Kramvis A, Dochez C, Meheus A. An update after 16 years of hepatitis B vaccination in South Africa. Vaccine. 2012;30 Suppl 3:C45-51. Epub 2012/09/04. doi: 10.1016/j.vaccine.2012.02.021. PubMed PMID: 22939021.
8. Bwogi J, Braka F, Makumbi I, Mishra V, Bakamutumaho B, Nanyunja M, et al. Hepatitis B infection is highly endemic in Uganda: findings from a national serosurvey. Afr Health Sci. 2009;9(2):98-108. PubMed PMID: 19652743.
9. Kirk GD, Bah E, Montesano R. Molecular epidemiology of human liver cancer: insights into etiology, pathogenesis and prevention from The Gambia, West Africa. Carcinogenesis. 2006;27(10):2070-82. doi: 10.1093/carcin/bgl060. PubMed PMID: 16679307.
10. Mendy M, Peterson I, Hossin S, Peto T, Jobarteh ML, Jeng-Barry A, et al. Observational study of vaccine efficacy 24 years after the start of hepatitis B vaccination in two Gambian villages: no need for a booster dose. PLoS One. 2013;8(3):e58029. doi: 10.1371/journal.pone.0058029. PubMed PMID: 23533578; PubMed Central PMCID: PMCPMC3606345.
11. van der Sande MA, Waight PA, Mendy M, Zaman S, Kaye S, Sam O, et al. Long-term protection against HBV chronic carriage of Gambian adolescents vaccinated in infancy and immune response in HBV booster trial in adolescence. PLoS One. 2007;2(8):e753. doi: 10.1371/journal.pone.0000753. PubMed PMID: 17710152; PubMed Central PMCID: PMCPMC1940311.
12. Whittle H, Jaffar S, Wansbrough M, Mendy M, Dumpis U, Collinson A, et al. Observational study of vaccine efficacy 14 years after trial of hepatitis B vaccination in Gambian children. BMJ. 2002;325(7364):569. PubMed PMID: 12228132; PubMed Central PMCID: PMCPMC124550.
13. Wright CM, Boudarene L, Ha NT, Wu O, Hawkins N. A systematic review of hepatitis B screening economic evaluations in low- and middle-income countries. BMC Public Health. 2018;18(1):373. doi: 10.1186/s12889-018-5261-8. PubMed PMID: 29558894; PubMed Central PMCID: PMCPMC5859762.
