# Supplementary material for: Hepatitis B virus seroepidemiology data for Africa: Modelling intervention strategies based on a systematic review and meta-analysis
Source: PLoS Med. 2020 Apr 21;17(4):e1003068. doi: 10.1371/journal.pmed.1003068 (PMC7173646; doi:10.1371/journal.pmed.1003068)
Supplement: S1 Fig — Confidence intervals for the population mean were calculated as x-±t′Sn, in which S is the standard deviation, x- the sample mean, n the cohort size, and t’ the upper (1 − C)/2 critical value for the t distribution with n − 1 degrees of freedom. Panels (a) for anti-HBc, (b) for HBsAg, and (c) for susceptible population. Full metadata online at Figshare: 10.6084/m9.figshare.6154598. (PDF) [file pmed.1003068.s006.pdf]

S1 Fig: Forest plots of HBsAg and anti-HBc prevalence and proportion of the population remaining susceptible in all studies identified in Africa (1995 – 2019). Confidence intervals (CI) for the population mean were calculated as $\bar{x} \pm t' S/\sqrt{n}$, where S is the standard deviation, $\bar{x}$ the sample mean (S2 Table), n the cohort size, and t' the upper (1-C)/2 critical value for the t distribution with n-1 degrees of freedom. Panels (a) for anti-HBc, (b) for HBsAg and (c) for susceptible population.

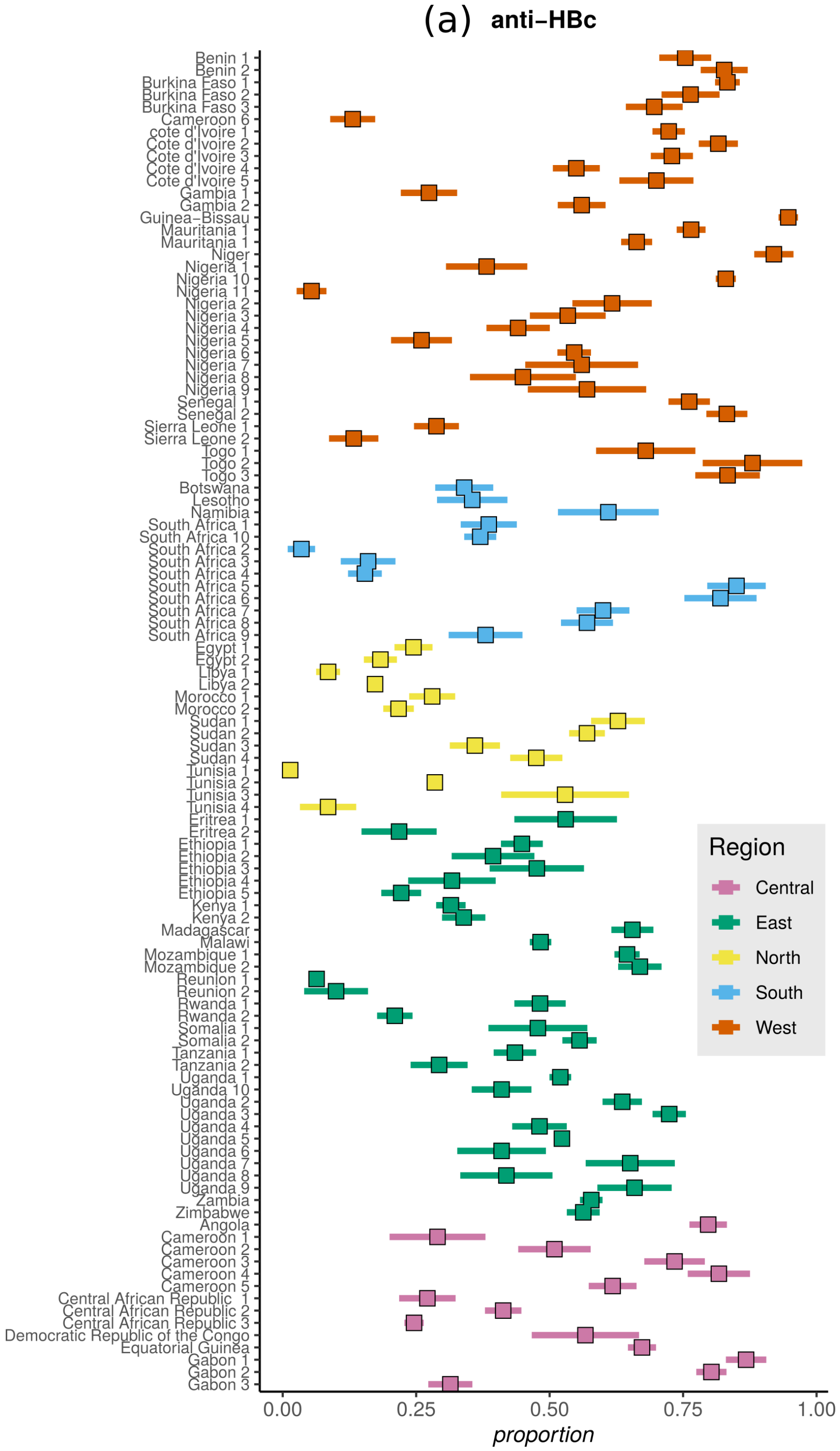

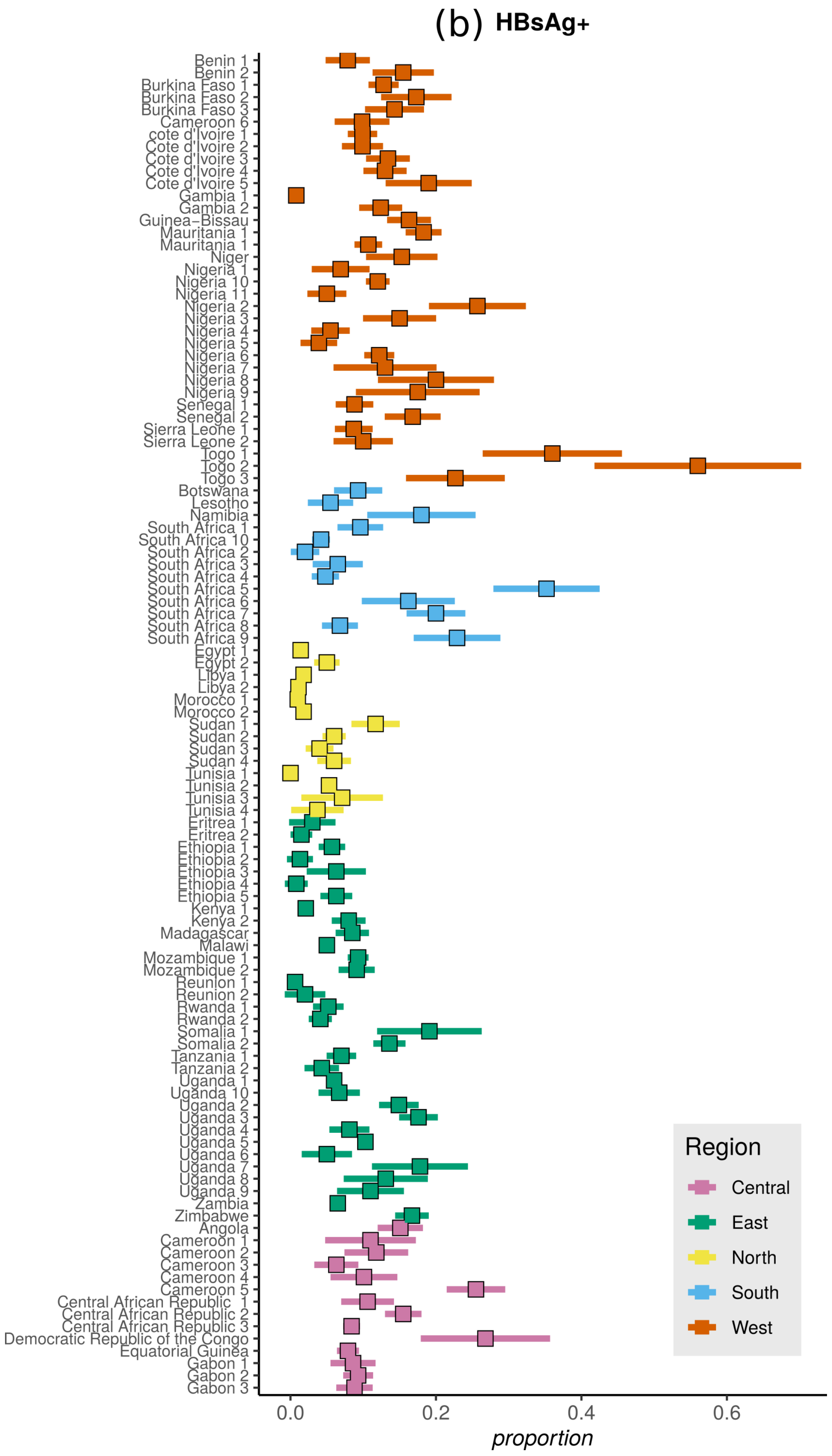
(b) HBsAg+
Benin 1
Benin 2
Burkina Faso 1
Burkina Faso 2
Burkina Faso 3
Cameroon 6
cote d'Ivoire 1
Cote d'Ivoire 2
Cote d'Ivoire 3
Cote d'Ivoire 4
Cote d'Ivoire 5
Gambia 1
Gambia 2
Guinea-Bissau
Mauritania 1
Mauritania 1
Niger
Nigeria 1
Nigeria 10
Nigeria 11
Nigeria 2
Nigeria 3
Nigeria 4
Nigeria 5
Nigeria 6
Nigeria 7
Nigeria 8
Nigeria 9
Senegal 1
Senegal 2
Sierra Leone 1
Sierra Leone 2
Togo 1
Togo 2
Togo 3
Botswana
Lesotho
Namibia
South Africa 1
South Africa 10
South Africa 2
South Africa 3
South Africa 4
South Africa 5
South Africa 6
South Africa 7
South Africa 8
South Africa 9
Egypt 1
Egypt 2
Libya 1
Libya 2
Morocco 1
Morocco 2
Sudan 1
Sudan 2
Sudan 3
Sudan 4
Tunisia 1
Tunisia 2
Tunisia 3
Tunisia 4
Eritrea 1
Eritrea 2
Ethiopia 1
Ethiopia 2
Ethiopia 3
Ethiopia 4
Ethiopia 5
Kenya 1
Kenya 2
Madagascar
Malawi
Mozambique 1
Mozambique 2
Reunion 1
Reunion 2
Rwanda 1
Rwanda 2
Somalia 1
Somalia 2
Tanzania 1
Tanzania 2
Uganda 1
Uganda 10
Uganda 2
Uganda 3
Uganda 4
Uganda 5
Uganda 6
Uganda 7
Uganda 8
Uganda 9
Zambia
Zimbabwe
Angola
Cameroon 1
Cameroon 2
Cameroon 3
Cameroon 4
Cameroon 5
Central African Republic 1
Central African Republic 2
Central African Republic 3
Democratic Republic of the Congo
Equatorial Guinea
Gabon 1
Gabon 2
Gabon 3
0.0
0.2
0.4
0.6
proportion
Region
Central
East
North
South
West

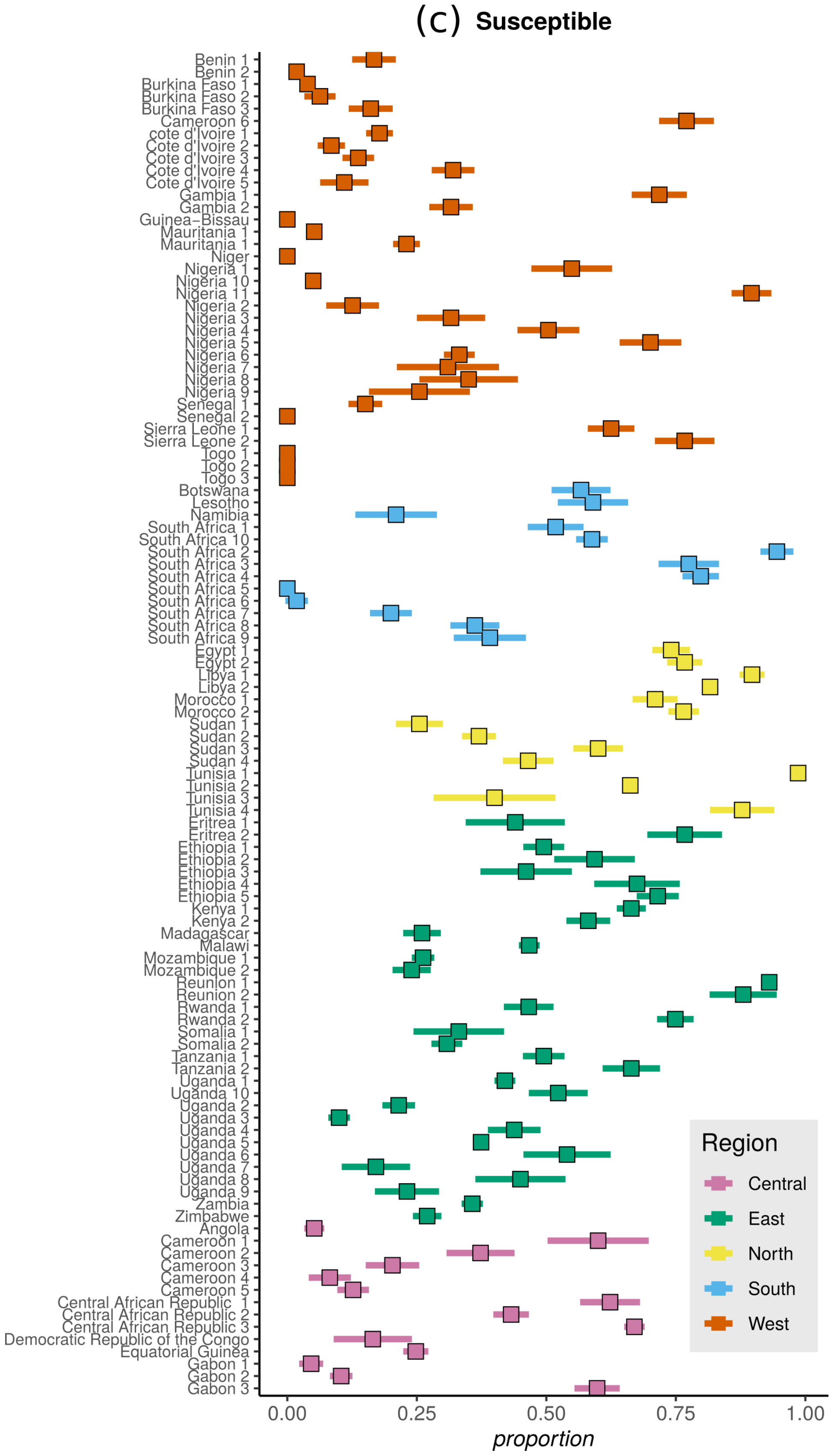
(c) Susceptible
Benin 1
Benin 2
Burkina Faso 1
Burkina Faso 2
Burkina Faso 3
Cameroon 6
cote d'Ivoire 1
Cote d'Ivoire 2
Cote d'Ivoire 3
Cote d'Ivoire 4
Cote d'Ivoire 5
Gambia 1
Gambia 2
Guinea−Bissau
Mauritania 1
Mauritania 1
Niger
Nigeria 1
Nigeria 10
Nigeria 11
Nigeria 2
Nigeria 3
Nigeria 4
Nigeria 5
Nigeria 6
Nigeria 7
Nigeria 8
Nigeria 9
Senegal 1
Senegal 2
Sierra Leone 1
Sierra Leone 2
Togo 1
Togo 2
Togo 3
Botswana
Lesotho
Namibia
South Africa 1
South Africa 10
South Africa 2
South Africa 3
South Africa 4
South Africa 5
South Africa 6
South Africa 7
South Africa 8
South Africa 9
Egypt 1
Egypt 2
Libya 1
Libya 2
Morocco 1
Morocco 2
Sudan 1
Sudan 2
Sudan 3
Sudan 4
Tunisia 1
Tunisia 2
Tunisia 3
Tunisia 4
Eritrea 1
Eritrea 2
Ethiopia 1
Ethiopia 2
Ethiopia 3
Ethiopia 4
Ethiopia 5
Kenya 1
Kenya 2
Madagascar
Malawi
Mozambique 1
Mozambique 2
Reunion 1
Reunion 2
Rwanda 1
Rwanda 2
Somalia 1
Somalia 2
Tanzania 1
Tanzania 2
Uganda 1
Uganda 10
Uganda 2
Uganda 3
Uganda 4
Uganda 5
Uganda 6
Uganda 7
Uganda 8
Uganda 9
Zambia
Zimbabwe
Angola
Cameroon 1
Cameroon 2
Cameroon 3
Cameroon 4
Cameroon 5
Central African Republic 1
Central African Republic 2
Central African Republic 3
Democratic Republic of the Congo
Equatorial Guinea
Gabon 1
Gabon 2
Gabon 3
0.00
0.25
0.50
0.75
1.00
proportion
Region
Central
East
North
South
West
