# Supplementary material for: Hepatitis B virus seroepidemiology data for Africa: Modelling intervention strategies based on a systematic review and meta-analysis
Source: PLoS Med. 2020 Apr 21;17(4):e1003068. doi: 10.1371/journal.pmed.1003068 (PMC7173646; doi:10.1371/journal.pmed.1003068)

S2 Fig: Average prevalence of anti-HBc and HBsAg in confirmed HIV-positive cohorts and all other cohorts. Cohort characteristics were recorded for each study (S2 table). All cohorts characterised as HIV-positive (n=27) were grouped together and compared with cohorts that were not listed at being HIV positive (n=79). Three cohorts testing sex workers (n=1) and patients in HIV testing clinics (n=2) were excluded from the analysis as not all subjects were HIV positive in these cohorts, but HIV positive subjects are likely to be enriched in these cohorts. Weighted averages, accounting for study size are shown along with 95% confidence intervals. No significant differences were identified for either anti-HBc or HBsAg prevalence (both p=0.06 and p=0.07 respectively).

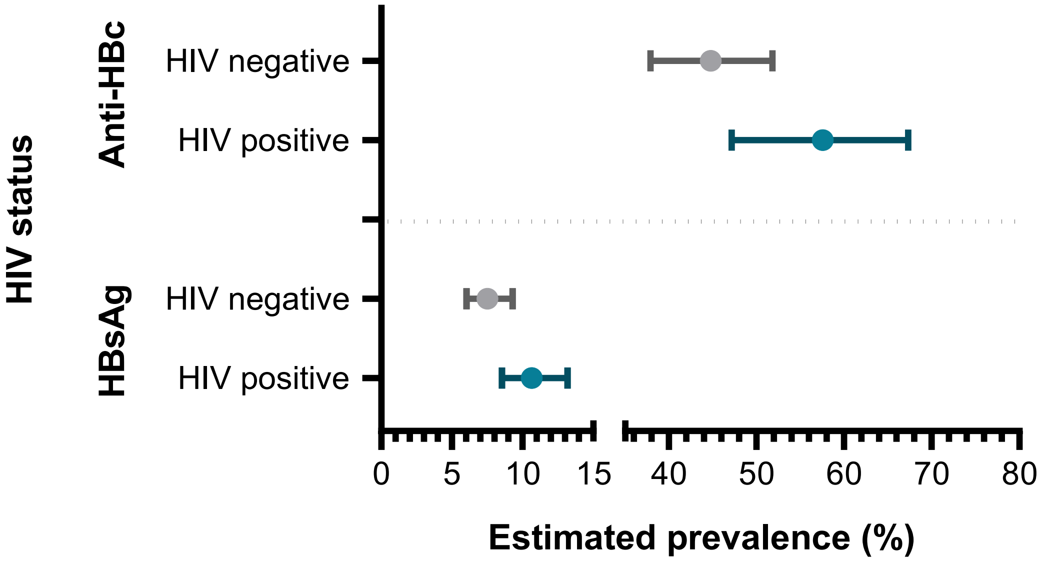

Supplement: S2 Fig — Cohort characteristics were recorded for each study (full metadata online at Figshare: 10.6084/m9.figshare.6154598). All cohorts characterised as HIV positive (n = 27) were grouped together and compared with cohorts that were not listed as being HIV positive (n = 79). Three cohorts testing sex workers (n = 1) and patients in HIV testing clinics (n = 2) were excluded from the analysis as not all participants were HIV positive in these cohorts, but HIV-positive participants are likely to be enriched in these cohorts. Weighted averages, accounting for study size, are shown along with 95% confidence intervals. No significant differences were identified for either anti-HBc or HBsAg prevalence (both p = 0.06 and p = 0.07, respectively). (PDF) [file pmed.1003068.s007.pdf]
