# Supplementary material for: Hepatitis B virus seroepidemiology data for Africa: Modelling intervention strategies based on a systematic review and meta-analysis
Source: PLoS Med. 2020 Apr 21;17(4):e1003068. doi: 10.1371/journal.pmed.1003068 (PMC7173646; doi:10.1371/journal.pmed.1003068)

S3 Fig: Predicted HBsAg prevalence for Northern, Eastern, Southern, Western and Central regions of Africa with a given total anti-HBc prevalence (reflecting exposure). Weighted linear regression (WLR) was performed using cohort size as weight. Predicted HBsAg prevalence by WLR for Northern, Eastern, Southern, Western and Central Africa at anti-HBc prevalences ranging from 5-95%, increasing in increments of 5% is presented here. Plotted from values given in S3 Table, data points and variation in Fig 4 of the main text.

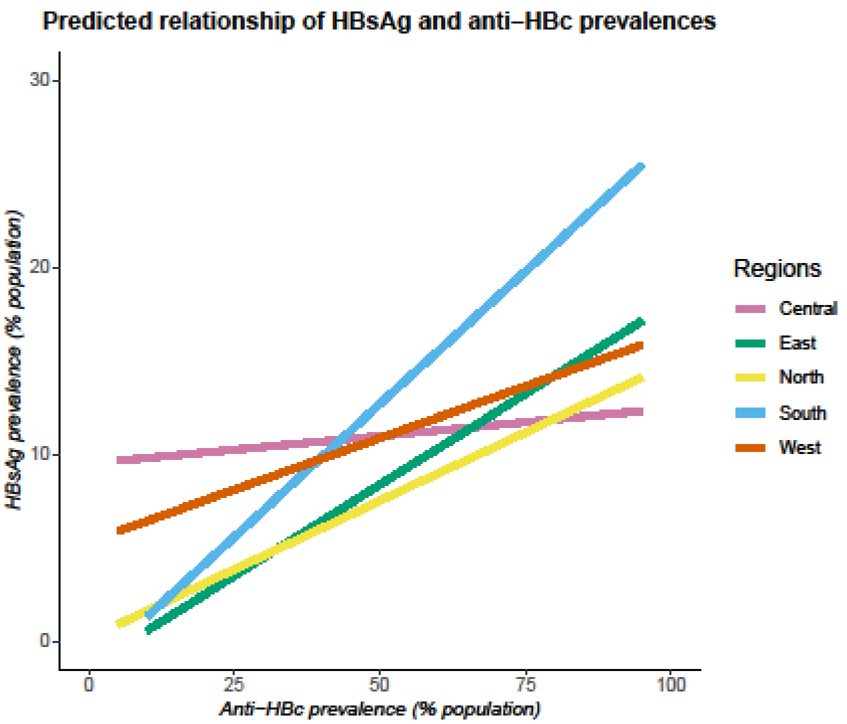

Supplement: S3 Fig — WLR was performed using cohort size as weight. Predicted HBsAg prevalence by WLR for Northern, Eastern, Southern, Western, and Central Africa at anti-HBc prevalences ranging from 5% to 95%, increasing in increments of 5%, is presented here. Plotted from values given in S3 Table. WLR, weighted linear regression. (PDF) [file pmed.1003068.s008.pdf]
