# Supplementary material for: Hepatitis B virus seroepidemiology data for Africa: Modelling intervention strategies based on a systematic review and meta-analysis
Source: PLoS Med. 2020 Apr 21;17(4):e1003068. doi: 10.1371/journal.pmed.1003068 (PMC7173646; doi:10.1371/journal.pmed.1003068)
Supplement: Attachment — Submitted filename: PMEDICINE D -19-03078 Manuscript review (1) (1).docx [file pmed.1003068.s009.docx]

Manuscript review

**Title: HBV seroepidemiology data from Africa provides insight into transmission and prevention.**

The need to scale up effective intervention in the viral hepatitis response in Africa is important and timely as noted in the manuscript. The article evaluates the relationship between HBV prevalence (HBsAg) and exposure (anti-HBc) in order to provide insight into HBV seroepidemiology and inference for the effectiveness of several interventions (i.e. preventive vaccination vs test and treat strategy). The authors also deploy modelling (previously published dynamic model) over a two-hundred year (200 year) period to show that HBV Test and Treat (as done in HIV) has a marked impact on reducing prevalence, equivalent to that of infant immunization.

The methodology (systematic review for serology and modelling for impact), results and discussion are well noted and the relationship between prevalence and exposure is intriguing. The impact modelling for 200 years is subject to an unpredictable future time period.

2 main areas for further clarity and/or improvement

a) The inclusion of HBV exposure as expressed by the presence of anti-HBc is noted to be an important advantage for this manuscript. However, tests for anti-HBc are subject to significant variation in sensitivity and specificity (Rapid tests, ELIZA etc) making interpretation and inference about anti-HBc prevalence challenging. The Inclusion criteria does not specify clear selection for study or test quality (lines 140-146) -this should be highlighted in study limitations.
b) The testing & treatment model is based on universal treatment of all HBsAg positive persons (lines 362 & 363) with the potential to treat many individuals who do not meet current criteria on regional or international treatment guidelines. As the article highlight the considerable gains that could be made through systematic deployment of universal T&T Lines 326) it is also important to highlight the potential harms of the strategy including treatment adherence issues and potential development of drug resistance.
c) The relevance of modelling for 200 to 500 years to reach conclusion may be subjective, is unclear and would definitely benefit from additional review by an expert in this area
