# Supplementary material for: Hepatitis B virus seroepidemiology data for Africa: Modelling intervention strategies based on a systematic review and meta-analysis
Source: PLoS Med. 2020 Apr 21;17(4):e1003068. doi: 10.1371/journal.pmed.1003068 (PMC7173646; doi:10.1371/journal.pmed.1003068)
Supplement: Attachment — Submitted filename: 191128 T4P response to reviewers.docx [file pmed.1003068.s010.docx]

**RESPONSE TO REVIEWERS**

**Ref: Manuscript "HBV seroepidemiology data for Africa provides insights into transmission and prevention" (PMEDICINE-D-19-03078)**

We are grateful to the editorial team and reviewers for providing detailed and thoughtful feedback on our paper. We were particularly pleased to have such positive feedback, including strong endorsement of the paper as a ‘well-written, excellent, comprehensive and creative article’ and ‘Thanks very much for giving me the opportunity to review your excellent paper’. We have addressed each individual point in turn, and our response to each individual comment is highlighted in yellow below.

1. **EDITORIAL COMMENTS**

We ask every co-author listed on the manuscript to fill in a contributing author statement.

We understand that this has already been completed by most of our authors at the time of the initial submission, the exceptions being José Lourenço, Sheila Lumley, and Tongai Maponga. We will notify them that they will be re-sent the authorship confirmation letter upon resubmission to make sure they fill it in.

In addition, we request that you upload any figures associated with your paper as individual TIF or EPS files with 300dpi resolution at resubmission.

Figures have been provided accordingly.

Deposit your laboratory protocols in protocols.io, where a protocol can be assigned its own identifier (DOI) such that it can be cited independently in the future.

We have not used laboratory protocols in this paper, so have not made any specific deposit. However, all modelling tools we used for analysis are published, either as a component to this manuscript, or in the previous modelling paper on which this latest analysis builds.

**Specific changes to the manuscript:**

1- Please report your SR/MA according to the PRISMA guidelines provided at the EQUATOR site.<http://www.equator-network.org/reporting-guidelines/prisma/>. Please provide the completed PRISMA checklist. When completing the checklist, please use section and paragraph numbers, rather than page numbers.

We have completed the PRISMA checklist; this is attached to the submission.

2- Please revise your title according to PLOS Medicine's style. Your title must be nondeclarative and not a question. It should begin with main concept if possible. "Effect of" should be used only if causality can be inferred, i.e., for an RCT. Please place the study design ("A randomized controlled trial," "A retrospective study," "A modelling study," etc.) in the subtitle (ie, after a colon).

The original title of the manuscript was ‘HBV seroepidemiology data for Africa provides insights into transmission and prevention’. We have modified this as requested; the new title is ‘Hepatitis B virus seroepidemiology data for Africa: a systematic review and meta-analysis to inform analysis of optimal local intervention strategies’. The new title incorporates the methodology we have employed, but also highlights the importance of analysis beyond just a review and meta-analysis, and underlines the translational influence of the results.

3- Please structure your abstract using the PLOS Medicine headings (Background, Methods and Findings, Conclusions).

We have changed this accordingly.

4- Please remove the third sentence of the Abstract (“HBV has been neglected...”) .

Done as requested.

5- In the Abstract Methods and Findings, please include information on the modelled population and timeframe for the estimates, and main outcome measures. For the systematic review portion, please provide the dates of search, data sources, number of studies included, types of study designs included, eligibility criteria, and synthesis/appraisal methods.

We have modified the abstract to include the following:

- Date of systematic literature search stated as November 2019;
- Dates of data included in the systematic review (publications reporting data collected between 1995-2019);
- Sources of data are stated as PubMed and Web of Science;
- We have summarised inclusion/eligibility criteria as ‘studies of adults that reported prevalence of both HBsAg (prevalence of HBV infection) and anti-HBc (prevalence of HBV exposure)’ between the specified dates;
- Number of studies included (96 studies representing 39 African countries);
- We have included details of methods for analysis (including the mathematical model and weighted linear regression analysis);
- Main outcome measures include: the strong relationship between the prevalence of HBsAg and anti-HBc (p<0.0001), and the impact of T&T intervention ‘predicted reduction of 33% prevalence by 20 years, and 62% at 50 years (at 100% coverage)’.

6- In the Abstract Methods and Findings, please quantify the main results (with 95% CIs and p values).

Where appropriate, we have included statistical results in the abstract.

7- Line 70 and throughout: Unless there is a statistical reason to do so, please report p values down to p= 0.001 and anything smaller as p<0.001.

Done as requested

8- In the last sentence of the Abstract Methods and Findings section, please describe the main limitation(s) of the study's methodology.

We have added as follows: ‘The study is constrained by gaps in the published data, such that we could not model the impact of antiviral therapy based on stratification by specific clinical criteria and our model framework does not include explicit age-specific or risk-group assumptions regarding force of transmission’.

9- At this stage, we ask that you include a short, non-technical Author Summary of your research to make findings accessible to a wide audience that includes both scientists and non-scientists. The Author Summary should immediately follow the Abstract in your revised manuscript. This text is subject to editorial change and should be distinct from the scientific abstract. Please see our author guidelines for more information: <https://journals.plos.org/plosmedicine/s/revising-your-manuscript#loc-author-summary>

We have added a non-technical Author Summary as requested.

10- Please remove the last sentence of the Introduction (“Our results have immediate potential…”).

Done as requested.

11- Please include the PRISMA flowchart in the main paper.

We have generated a new PRISMA figure which is now presented as Fig 1 in the final manuscript, together with a supporting figure legend.

12- Please update your literature search to the present time.

We have updated the literature review, identifying 9 new studies that we have incorporated into the analysis, and updating the abstract and methods to report the time period represented by our literature review. The opening sentence of the results section (page 13) is updated to read: ‘We collated and curated a total of 96 studies spanning 39 African countries and generating 109 unique data points’, and these numbers are also presented in the PRISMA flow chart (Fig 1).

13- As noted by reviewer 1, please conduct a meta-analysis.

We have formalised the meta-analysis component of the study, repeating the literature review to bring it up to date, and weighting the analysis to account for study size. More specific details of the new approach and updated figures are included in our specific responses to reviewer 1, below. In order to undertake appropriate statistical analysis, we have needed to enlist additional expert help, and have added Uri Obolski as an author to reflect his significant contributions to the new analysis that was requested.

14- Please evaluate study quality and risk of bias.

We have undertaken a formal appraisal of study quality and bias using a standardised tool from the Joanna Briggs institute, JBI (<https://joannabriggs.org/>). To support this approach, we have added a citation to the JBI checklist, and have references the corresponding peer reviewed publication (Munn Z, et al., Int J Evid Based Healthc. 2015;13(3):147–153). The output of our quality appraisal is included in a new suppl data cited and available on-line together with our main study metadata (Figshare https://figsharecom/s/4414fce1d474bc8a6198 [DOI 106084/m9figshare6154598]). We have also added a section to the results, entitled ‘Quality appraisal of data identified through systematic review of the literature’ (page 13), setting out the findings from our reassessment of all the papers included in our review. We have also added a reference from the JBI to highlight the importance of disseminating this data for Africa to the discussion (page 17): ‘This is all the more important for resource-limited settings that are under-represented in the existing evidence base (Pearson et al., Int J Evid Based Healthc. 2010 Jun;8(2):97-100).

15- Though the HBV dynamic model was previously published, please provide more details on the model in the Methods section.

The new Figure 2 sets out the model classes and dynamic structure of the model (also discussed in point 16, below), with supporting details of each class provided in the figure legend. We have also added more details to the methods section of the paper, under the heading ‘*Modelling the impact of adult vaccination vs. ‘test and treat’* (page 12).

16- Please provide a diagram that shows the model structure, including how the disease natural history is represented, the process and determinants of disease acquisition, and how the putative intervention could affect the system.

We have generated a flow chart to show the classes represented by the model, including representation of the points where interventions take effect (new Figure 2). Please note that the full model is already published (McNaughton et al., BMC Medicine 2019, doi: 10.1186/s12916-019-1269-x), so we have not replicated a diagram showing all the parameters, but have provided enough information to allow readers to understand the model classes and interventions without having to refer to the other publication.

17- What were the algorithms used for HBsAg and anti-HBc in the various studies?

Typically, an algorithmic approach is not used to measure or interpret either of these serological assays - so we are uncertain exactly what is being asked for.

We recognise that different studies may have used different assays to measure these parameters, and have added to the limitations and caveats section to make this explicit, including a supporting reference (Coffin C. et al., Gastroenterology 2019;156:355–368). We have also included assessment of completeness of reporting of the laboratory methods in each individual study within the quality appraisal of the literature review (suppl table xxx).

If any further clarification or amendment is required, please let us know and we will be happy to consider additional modifications.

18- Does the model take into account non-compliance with drug treatment?

Fig 6E shows the impact of less than 100% coverage with drug therapy, which demonstrates the impact of different proportions of the population who are not taking treatment (whether a result of not being treated or not taking treatment). We have amended the figure legend to incorporate this point: ‘This demonstrates the impact of reduced proportions of the population exposed to drug therapy, either as a result of not having drug prescribed, or non-compliance with prescribed therapy.’

19- Please use the "Vancouver" style for reference formatting, and see our website for other reference guidelines <https://journals.plos.org/plosmedicine/s/submission-guidelines#loc-references>. Reference 4, for example, is incomplete.

We have reformatted in Vancouver style and updated ref 4.

**Comments from the reviewers:**

**Reviewer #1: Alex McConnachie, Statistical Review**

McNaughton et al look at transmission and prevention of HBV in Africa; this review looks at the statistical aspects of the paper.

1. The paper involves a systematic review, but no meta-analysis. At least, it is not described as such; the analysis of metadata is done with equal weight given to each study, and groups of studies are compared with standard independent samples tests. This seems wrong - studies should be weighted according to their size, or the precision with which they estimate prevalence.

We have updated the paper, with the help of a new expert statistical author (Dr Uri Obolski) to incorporate a weighted meta-analysis of the data, based on the size of each study (section headed ‘statistical analysis of metadata’ on page 11). This new analysis is incorporated in the updated version of Figs 4 and 5, and is also supported by the figure legends.

2. The phrase "non-parametric data" is not correct. It is the test used to analyse the data that is non-parametric, not the data itself. Also, if a non-parametric test is used to compare two groups, then ANOVA for comparing more than two groups is not right; something like a Kruskal-Wallis test would seem appropriate.

We have removed the term non-parametric data, and have updated our statistical methods on page 12 as follows: ‘Estimates of pooled median proportions (HBsAg+, anti-HBc), were obtained through logistic regression with a random intercept assigned to each cohort, performed separately for each African region. The 95% confidence intervals of the proportions were based on profile likelihood of the model’s fixed effect intercepts. Comparisons between different regions were obtained by subsetting the data for each pair of regions and creating two models. The first was the logistic regression model with a random intercept assigned to each cohort described above, while the second had an added fixed effect of the study region. These two models were compared through the likelihood ratio test (LRT). The obtained p-values from the LRTs were then adjusted for multiple comparisons using the Holm-Bonferroni method, and the adjusted p-values are reported’.

3. The paper then describes the results of adapting a model they have built to explore the impact of adult vaccination, or a Test and Treat approach on HBV control. The model is applied using data obtained from the systematic review specifically from Uganda. The results, shown in Figure 4, appear to include various other strategies, which are not well described in the paper.

Other strategies shown in this figure (originally Fig 4, now Fig 6) have been previously described and evaluated in detail by our earlier paper - to make this clearer, we have added this point into the figure legend with the supporting reference (McNaughton et al., BMC Medicine 2019, doi: 10.1186/s12916-019-1269-x). It would be inappropriate to duplicate previous analysis, but we are confident that the new figure showing the structure of the model (Fig 2), together with additional explanation of the model in the methods of the paper, provide a better foundation for understanding these other strategies.

4. Also, I do not see the value of projecting the impact of strategies up to 500 years into the future. Even a 50-year horizon is probably beyond most policy-makers considerations.

This issue has also been raised by other reviewers, and we completely agree that the long time scales over which we had projected are not useful in terms of informing policy, so we have amended the figures accordingly. Specifically, Figure 6 now shows timelines of 50 years for interventions (panels A-E). However, our original rationale for the long time periods was to ilustrate the projected timelines required to reach extinction; for this reason we have also included a final panel (Fig 6F) showing the longer time line.

5. No attempt appears to have been made to explore the potential impact of different control strategies in different settings. E.g. are there any settings in which a catch-up vaccination strategy would be effective, or in which a T+T strategy less so? There is some speculation about this in the discussion, but it would be better to use the model to investigate what the optimal strategies might be in different settings.

We agree that this is a valuable use of the model and future data, and represents the foundation of a new project we are now working on. However these are very substantial questions that require careful exploration, evaluation and new analysis. We have now expanded this current paper to report a full systematic review and meta-analysis (including nearly 30 pages of suppl data!), so to add multiple more modelling questions is not possible or appropriate within sensible space constraints. For these reasons, we would prefer to constrain the current manuscript to the hypothesis and evaluation we set out to deliver, and to explore these follow-up questions in future analyses.

To reflect this interesting discussion, we have modified the final paragraph of the paper by adding ‘future investigation’ into one of our subheadings and adding the following text: ‘Further analysis is required to investigate more specifically the populations or contexts in which vaccination strategies or T&T might best be deployed’.

Please note that we would be happy to discuss this further with the reviewer or editorial team if concerns remain that this is an essential component of revisions to the current manuscript.

**Reviewer #3:** This manuscript describes a systematic review on the prevalence of chronic HBV and modelled estimates of the impact of catch up vaccination or test and treat in Africa. The results suggest heterogenous prevalence of chronic HBV with regional variation. Catch-up vaccination has predictably little impact on chronic HBV prevalence. Test & Treat appears to be an effective method for reducing the prevalence of chronic infection albeit that the impact will take some time to achieve SDG goals.

1. The wording of the abstract is a little confusing - "relationship between the prevalence of HBsAg and anti-HBc (p<0.0001)" All patients who are HBsAg positive are also anti-HBc positive. It would help if the authors made it clearer that they are talking about prevalence of chronic infection and previous acute infection (exposure)

We agree this is potentially ambiguous, and have double-checked this point throughout the manuscript to add clarity. In fact, anti-HBc is generally reported in the literature (and therefore assessed by us) as *total* exposure (including both current and previous infection). In the methods, we have included careful definitions to avoid confusion: ‘We recorded total anti-HBc prevalence (i.e. proportion of population exposed to HBV, irrespective of chronic infection status, termed ‘total exposure’) and also calculated the proportion of the population with cleared infection (i.e. anti-HBc prevalence minus HBsAg prevalence, termed ‘exposed and cleared’)’. In the legends for Fig 4 and Fig 5, we have amended to clarify ‘total exposure’ when using anti-HBc (ie this is the sum of the prevalence of active infection + those with serological evidence of previous exposure and clearance).

2. The term 'infection' is insufficiently precise. It is important to distinguish acute HBV infection from chronic HBV infection as the risk of cirrhosis and hepatocellular carcinoma are only associated with the latter

We have changed this throughout, including in the abstract, so that instead of referring to ‘infection’ we use ‘chronic hepatitis B infection’, and follow up with consistent use of the well accepted acronym, CHB (all relevant instances highlighted in tracked version of manuscript).

3. Whilst the authors are correct in asserting that catch-up vaccination in adolescents is still being proposed as a public health strategy, in reality this is now rarely advocated. It is well understood that 1. Exposure to the infection takes place earlier in life and 2. Exposure as an adolescent rarely results in chronic infection and is therefore a much less important public health issue. The only situation where catch up vaccination is potentially beneficial is in regions where HDV co-infection is prevalent and spread through sexual transmission

We agree that there are indeed striking discrepancies between what is advocated in published clinical practice guidelines, and policy that is being actively implemented in the field. One of the factors that inspired us to generate this study was the personal experience of seeing catch-up campaigns being actively deployed in Uganda. To ensure this discussion is represented as fully as possible in the manuscript, please note the following:

- Introduction: we have been careful to make sure we describe current recommendations clearly, so we say ‘There is an interesting conflict between formal recommendations, and public health policies that are deployed in practice’, and go on to explain that evidence for catch-up vaccination suggests ‘a limited population benefit and lack of endorsement by routine recommendations’ (page 9).
- We have cited a reference from the media to provide some (limited) evidence of catch-up vaccine campaigns - unfortunately, this is not well reported in the peer-reviewed literature so we cannot evidence it further. (Ref: Ampurire P. (<https://www.softpower.ug/hepatitis-b-vaccination-health-ministry-reports-significant-drop-in-coverage-of-follow-up-doses/> ). Soft Power News. 2019.)
- It is an interesting point that co-endemic HDV should be considered, and we have added this specifically to the discussion section (page 18) as follows: ‘...vaccination may be important in regions where HDV infection is highly prevalent’; this is supported by a new reference (Butler EK et al, High prevalence of hepatitis delta virus in Cameroon. Sci Rep. 2018;8(1):11617.’

4. The HBeAg positivity rate amongst those who are HBsAg positive is high (Suppl Table 1) . The rate will vary according to age and is most relevant in pregnant women.

We agree that the estimate for HBeAg prevalence will vary according to various parameters that may include genotype of infection, duration of infection, HIV status, host immune responses etc. The source we have used for HBeAg prevalence is Matthews et al, PLoS One. 2015;10(7):e0134037. As this represents HIV-positive cohorts, we agree this could be biased towards a high HBeAg prevalence (27%). In another recent study, we quote a comparable point estimate of 23% HBeAg prevalence in pregnant women, based on four studies but with wide confidence intervals of 16.7–42.9% (Mokaya et al [*BMC Public Health*](https://bmcpublichealth.biomedcentral.com/); <https://doi.org/10.1186/s12889-019-7095-4>). A prevalence of ~25% appears to be broadly representative, and making small changes to this parameter will not have an overall impact on output predictions from the model. We have added to the discussion, as follows: ‘Despite uncertainty in specific variables, the model fixes certain parameters, based on estimates from the literature (e.g. HBeAg is set at 27%, recognising that the confidence intervals around this point estimate are wide and vary between settings)’ (page 22).

5. Rate of HBeAg clearance uses an estimate from Taiwan rather than data from West Africa - see Shimakawa et al.

HBeAg clearance rates are difficult to ascertain from understudied populations in Africa, and for this reason we did not use a fixed parameter for this variable, but left it free (as indicated in suppl table 1 that lists parameters).

The reference we have cited in this suppl table (Kao JH, Hepatol Int. 2007;1(4):415-30) is simply to support the estimates that we generated from the model. It is of note that although the author of this paper is from Taiwan, and some of the data do focus on Taiwanese cohorts, the paper is a review that considers global patterns - so the estimated parameters are not Taiwan-specific. Kao’s paper estimates HBeAg loss at a rate of 0.8% a year for age <5 yrs; 3% for age 5-15 yrs; 8-15% for age >15 yrs. In the Shimakawa paper (Gut. 2016; doi:10.1136/gutjnl-2015-309892), the overall annual clearance rate of HBeAg was 7.4% ( 5.1% a year age 0-9 yrs; 8.7% for age 10-19 yrs; 8.6% for age ≥20 yrs). The output from our model reassuringly recovers rates for this parameter that overlap with those that have been published by both Kao and Shimakawa - with an aggregate estimate of 5.12% (range 4.2-6.4%) across all ages.

6. The paper does not seem to take into account current indications for treatment. If advocating treatment for all patients with chronic infection irrespective of disease phase, level of viraemia etc then this needs discussing in more detail.

Based on this feedback, we have added a new paragraph into the introduction (pages 8-9) describing the use of NA agents and the specific description of treatment algorithms (as also suggested by Reviewer 4). We have included references to treatment recommendations published by WHO, EASL, NICE and South African National guidelines.

Our new text reads as follows: ‘Treatment of CHB is typically based on nucelos(t)ide analogue (NA) drugs, among which tenofovir disoproxil fumarate (TDF) is the most affordable, accessible and widely used. NA agents have a high rate of success in suppressing detectable viraemia, but do not tackle the liver reservoir of virus (in the form of covalently closed circular DNA in the nucleus of infected hepatocytes, so most individuals are committed to long-term treatment. Individuals with CHB are assessed for treatment based on algorithms that stratify the long term risk of liver disease based on age and sex, laboratory measurement of liver enzymes and HBV DNA viral load, elastography scores or other imaging data. Treatment guidelines also recognise and support the use of TDF during pregnancy as part of an approach to reduce vertical transmission, and this approach is widely accepted (albeit variably deployed)’.

We had previously tackled the interesting point about treatment for all patients with CHB in the brief discussion section entitled ‘‘Test and treat’ in the context of HBV guidelines’. In response to the suggestion that further details should be incorporated, we have expanded this section to recognise the risks/benefits associated with expanding access to treatment (as also suggested by reviewer 2). The complete paragraph now reads as follows:

‘Current guidelines for the management of CHB recommend treatment only in certain subgroups, based on risk of liver inflammation. Thus, deployment of a widespread T&T campaign would treat many individuals who do not meet current criteria. The infrastructure and budgets to support this detailed individual assessment are not available in many lower and middle income country settings, and there is a call for algorithms to be simplified. Relaxing the stringency of assessment for treatment may also have the added benefit of reducing complications from HBV progression, as not all individuals at high risk of cirrhosis or HCC are detected by current algorithms. Therefore, adopting a universal T&T strategy for HBV would need to be carefully evaluated to consider challenges and risks as well as benefits. Wider deployment of NA therapy involves a need for expanded infrastructure, imposes economic costs, and carries risks of drug side-effects and toxicity, as well as raising concerns about non-adherence and the associated potential for increasing the selection of drug resistance. However, these may be offset by the benefits of individual, societal and economic benefits of reducing the morbidity and mortality of chronic liver disease, as well as the population gains of minimising the risk of transmission’ (page 20).

7. Whilst the discussion encompasses consideration of exposure and chronic infection rate variation between African regions it missed the wide variation in prevalence and exposure at a much more granular level - see Whittle JID 1990

We have added this point to the first paragraph in the discussion, including addition of the suggested reference, expanding on our existing text in which we said ‘Notably, even with a single country – exemplified here by Uganda – we find evidence of region-specific differences in exposure, transmission and chronicity rates’, to include the higher resolution description by Whittle et al., ‘This is consistent with patterns previously described in the literature, with the potential for marked differences in sero-epidemiology even in neighbouring villages [ref: Whittle, J Infect Dis. 1990 Jun;161(6):1112-5]’.

8. I am not comfortable with postulating a role for insect vectors in HBV. This has been examined extensively and is not supported by evidence.

We have removed this from Table 1, together with the associated reference.

**Reviewer #4:**

Dear authors,

This is a well-written, excellent, comprehensive and creative article. The authors start with and support the hypothesis that testing and treating hepatitis B may be a viable public health option. The comprehensive literature review of hepatitis B serology and the modelling and compilation of data are clear and well-presented.

Thank you to reviewer 4 for this positive and enthusiastic feedback.

My comments concern the paper itself. There are three points that could be better introduced and discussed in the conclusions and recommendations, so that the appropriate focus is brought to the findings. An alternate presentation of data should be considered.

1. Firstly, the authors state at the outset that there are no recommendations for wide-scale catch-up immunization of adults. Yet the scenario of a single one-off all ages catch-up vaccination campaign is one of the interventions modelled and studied in this paper. The authors then conclude that 'Although it can seem intuitive to deploy catch-up vaccination for adults in high prevalence settings... only a minority will benefit from the intervention'. Therefore they appear to advocate a blanket dismissal of 'catch-up' vaccination which they state is not actually the subject of any recommendation.

The reviewer is right about the conflict between concrete guidelines and practical interventions that are being deployed, and this has also been raised by another reviewer (see point 3 from reviewer 3, and our detailed responses set out above).

2. However the authors have not analyzed targeted vaccination strategies such as for example catch-up vaccination in low EPI coverage settings for young children to reduce childhood horizontal transmission, or assessed alternate booster/catch-up approaches in school-aged children prior to sexual début. So the emphasis on a non-recommended strategy risks detracting from consideration of possibly more useful vaccination and other alternatives. It would be far preferable for example to have the abstract, intro and discussion focus on the impact of vaccination at birth and/or after school entry as these are also modelled and far more relevant from a public health programmes perspective.

Several points are raised here, and we address them in turn:

***(i) ‘Useful’ vaccination strategies, including vaccination at birth***

Vaccination at birth is a standard approach that is already extremely well validated and explored, and is addressed by our earlier paper in which the mathematical model is published (McNaughton et al., BMC Medicine 2019, doi: 10.1186/s12916-019-1269-x). We agree this strategy is of fundamental importance, and have made some small modifications to make sure this is unambiguous, including adding to the abstract: ‘In African populations, infant immunisation has been fundamental to reducing incident infections in children…’, and to the discussion: ‘The importance of infant vaccination remains paramount, and has been well validated in clinical practice, mathematical models and population epidemiology across diverse settings’. The current paper also highlights the limitations to what can be achieved through ongoing pursuit of infant immunisation: ‘published mathematical models exploring the impact of existing interventions demonstrate time-scales for success that are substantially beyond the 2030 targets’.

***(ii) Emphasis on a ‘non-recommended strategy’***

The aim of this current manuscript is to evaluate different strategies that have *not* previously been robustly addressed, and to put a spotlight on poorly studied interventions in adults, which we have added more clearly into the final paragraph of the introduction, describing our focus on ‘...specific focus on interventions that target adults’, and modifying the discussion: ‘In this study, we set out to explore the controversial and less studied approaches of catch-up vaccination and T&T.’ Although catchup vaccination is not recommended, it is nevertheless being deployed in practice in the field (for more details, see our response to reviewer 3, point 3).

***(iii) Vaccination after school entry***

One of the interventions we have evaluated with our model is routine vaccination at school age (immunisation of all as they transit into age 6+ category) - as shown in Fig 2 and Fig 6B. This shows some impact of such a strategy, but less efficacy compared to other interventions.

3. The presentation of the models out to 500 years is nice but fanciful, and detracts from closer assessment of alternate strategies, and possible combinations of strategies, over the next ten to 50 years.

As per our feedback to reviewer 1, we have amended presentation of timelines to reflect the impact of interventions over a shorter time period. Figure 6 now primarily shows projections for 20 and 50 years, instead of 500.

4. My recommendation is to refocus the abstract, introduction and discussion on the range of possible public health interventions that should be considered, not on the one for which they found no recommendations.

We have carefully reviewed and updated the manuscript to reduce the extent to which we emphasise a specific spotlight on catch-up vaccination. We have instead shifted the focus slightly by explaining that we have considered two different population interventions that have not previously been evaluated in detail, comparing (i) catch-up vaccination, with (ii) test and treat (T&T). We then present results to show that catch-up has little impact, while T&T has the potential for substantial influence. The conclusions pertain most strongly to the gains that can be made through T&T.

Specifically, we have reviewed/amended the following sections:

- The new title now highlights ‘test and treat’ as a primary strategy for HBV
- Abstract (background section): is now modified to read as follows: ‘In high prevalence populations, adult catch-up vaccination has sometimes been deployed, or an alternative ‘test and treat’ (T&T) approach can be used as an intervention to interrupt transmission’.
- Abstract (conclusion section): we have reviewed and think that our existing statement that does indeed focus on the intervention which is most successful: ‘In combination with robust neonatal immunisation programmes, diagnosing and treating HBV infection is likely to be of most impact in driving advances towards elimination targets.’
- Discussion: we have improved the summary of our findings, by following the paragraphs about catch-up vaccination with a clearer link to the comparator strategy, T&T, drawing attention to this as a successful intervention with the linking sentence: ‘In contrast, our data suggest that T&T programmes aimed at older children and adults may have great impact…’.

5. The authors should consider also presenting findings to at most 50 or 100 years (or event 200, the relevance of which is also highly questionable) so that readers can really focus on the immediate public health implications of the paper. Highlighting the 2030 goal would add immediacy to the findings.

Please see our responses to point 3 above, and specifically the amendments to Fig 6. We have highlighted the 2030 goal in the opening sentence of the abstract, and in the second paragraph of the introduction. We also refer back to this in the legend of Fig 6, describing our analysis of ‘an overall time-frame that incorporates the 2030 elimination targets’.

6. I also recommend removing the data points for the one-off all ages vaccination campaign, as it also detracts from the far more relevant findings. Mentioning those results briefly in the text would be sufficient.

We are very keen to leave these data represented. While it is certainly true that this approach makes little impact at a public health level, one of the key attributes of this paper is to be able to point out to policy makers (who have been actively deploying catch-up campaigns) that this approach has no evidence base. Furthermore, this provides an important basis for the comparison with other interventions that do work well at a population level. Making this output accessible in a way that can be presented and accessed by a non-expert audience is very important in policy terms.

7. Secondly, the primary thesis of the paper concerns treatment of hepatitis B, yet there is no mention of treatment options upon which the paper relies. Please include a paragraph outlining the treatment options currently being used and some parameters considered (e.g. assumptions about what constitutes successful treatment or assumed success rates, mention of complications attributable to treatment). This does not have to be detailed, but sufficient to orient the reader to the public health intervention being proposed and argued for in this paper.

We have added a paragraph to the introduction to set the scene with more details of current treatment recommendations and outcomes. Please note we have also amended the discussion (in response to specific overlapping comments from reviewer 3), in order to reflect the risk/benefit appraisal of extending therapy to a wider proportion of the overall population with CHB.

8. Thirdly, the practical, logistical and economic feasibility of the T&T intervention is likely at this time to be quite low in the African continent and countries included in the study. The authors draw an analogy with HIV T&T, but there is no HIV vaccine, and those infected remain infectious and face morbidity and mortality much more quickly than chronic HPV carriers do, so it may be an inadequate comparison. The cost is likely to be prohibitive and the systems not in place. Perhaps cervical cancer screening would be a reasonable analogy. In order to orient the reader and provide more targeted recommendations, it would be helpful to provide any examples of where the T&T approach is being piloted for HBV and reference practical considerations.

We have amended the discussion paragraph entitled ‘‘Test and treat’ in the context of HBV guidelines’ to include the specific point about the substantial challenges that would be involved in wider deployment of T&T (page 20), with reference to specific practical considerations, saying: ‘Therefore, adopting a universal T&T strategy for HBV would need to be carefully evaluated to consider challenges and risks as well as benefits. Wider deployment of NA therapy involves a need for expanded infrastructure, imposes economic costs, and carries risks of drug side-effects and toxicity, as well as raising concerns about non-adherence and the associated potential for increasing the selection of drug resistance’.

We have also added a short section to the end of the paragraph entitled ‘Caveats and limitations’ (page 20) to recognise the imperfect use of HIV as a precedent, as follows: ‘While we have considered HIV as a precedent for T&T interventions, there are also important differences between HIV and HBV, namely the lack of an HIV vaccine, and the shorter average timeframes over which morbidity and mortality evolve in the setting of HIV infection.’

From a public health programming perspective and the 2030 targets, the most relevant question would be what would be the success of a primary prevention strategy combining 1.PMTCT 2.Routine neonatal HB vaccination and 3.School-based booster/catch-up HB vaccination. Then the question could be asked what is the marginal added value of a T&T intervention at scale. If this paper cannot address such questions now, then it would be helpful to set up the discussion to indicate what are the appropriate questions for public health programmes in Africa, so that subsequent research can pursue them.

We have added to the final paragraph of the discussion to set out the prospects for future research, with a new section subheading, and text that says: ‘Further analysis is required to investigate more specifically the populations or contexts in which vaccination strategies or T&T might best be deployed, and how the extent to which this intervention is synergistic with other routine interventions (infant vaccination and PMTCT).’.

Thanks very much for giving me the opportunity to review your excellent paper and I look forward to your further work.

Very kind, thank you for your helpful feedback!

**Reviewer 2? Manuscript review**

Title: HBV seroepidemiology data from Africa provides insight into transmission and prevention.

The need to scale up effective intervention in the viral hepatitis response in Africa is important and timely as noted in the manuscript. The article evaluates the relationship between HBV prevalence (HBsAg) and exposure (anti-HBc) in order to provide insight into HBV seroepidemiology and inference for the effectiveness of several interventions (i.e. preventive vaccination vs test and treat strategy). The authors also deploy modelling (previously published dynamic model) over a two-hundred year (200 year) period to show that HBV Test and Treat (as done in HIV) has a marked impact on reducing prevalence, equivalent to that of infant immunization. The methodology (systematic review for serology and modelling for impact), results and discussion are well noted and the relationship between prevalence and exposure is intriguing. The impact modelling for 200 years is subject to an unpredictable future time period.

2 main areas for further clarity and/or improvement

a) The inclusion of HBV exposure as expressed by the presence of anti-HBc is noted to be an important advantage for this manuscript. However, tests for anti-HBc are subject to significant variation in sensitivity and specificity (Rapid tests, ELIZA etc) making interpretation and inference about anti-HBc prevalence challenging. The Inclusion criteria does not specify clear selection for study or test quality (lines 140-146) -this should be highlighted in study limitations.

The addition of a specific quality assessment of the individual studies included in our review now provides improved insights into the reporting of laboratory testing that underpins the seroepidemiology data we report. We have added to the results with a new subsection (***Quality appraisal of data identified through systematic literature review***), to describe the output of this quality appraisal, including specific reference to the serological assays that have been employed:

‘The majority of studies included in the meta-analysis were of good quality, with 85% studies reporting clear details of the methods used to measure HBsAg and anti-HBc (Suppl Table 5), reflecting our stringent criteria for inclusion of these prevalence values’.

We have also amended the discussion to incorporate recognition of the caveats of pooled serology data, which now reads ‘The sensitivity and specificity of serological assays vary, which may represent a source of bias or uncertainty in our dataset’, together with a reference to Coffin et al. (Gastroenterology. 2019;156(2):355-68 e3), which reviews this issue in more detail.

b) The testing & treatment model is based on universal treatment of all HBsAg positive persons (lines 362 & 363) with the potential to treat many individuals who do not meet current criteria on regional or international treatment guidelines. As the article highlight the considerable gains that could be made through systematic deployment of universal T&T Lines 326) it is also important to highlight the potential harms of the strategy including treatment adherence issues and potential development of drug resistance.

This point overlaps substantially with points raised by reviewers 3 and 4, both of whom also suggested adding more details about the nature and impact of treatment (now included in the introduction) and the implications of suggesting wider deployment of therapy (now added to the discussion). In the discussion section, we have specifically mentioned harms including treatment adherence and drug resistance. The specific new text sections are included in our responses to earlier reviews within this document.

c) The relevance of modelling for 200 to 500 years to reach conclusion may be subjective, is unclear and would definitely benefit from additional review by an expert in this area

As per comments raised by reviewers 1 and 4, we have reduced the timelines to focus on a shorter time period; please see our feedback to their comments for a more detailed summary of the specific changes we have made.
